# Lower-dose corticosteroid therapy in severe immune thrombocytopenia during pregnancy: The comparable efficacy and lower incidence of maternal complications

**DOI:** 10.3389/fphar.2022.983734

**Published:** 2022-10-20

**Authors:** Xue Xu, Mei-Ying Liang, Yi-Lin Wang, Jian-Liu Wang, Xiao-Hui Zhang

**Affiliations:** ^1^ Obstetrics and Gynecology, Peking University People’s Hospital, Beijing, China; ^2^ Hematology, Peking University People’s Hospital, Beijing, China

**Keywords:** pregnancy, immune thrombocytopenia, corticosteroid therapy, efficacy, maternal and neonatal outcomes

## Abstract

**Background:** This study assessed the clinical efficacy of oral prednisone at low dose (LD) *versus* the previous high-dose (HD) study in patients with severe immune thrombocytopenia during pregnancy and its side effects on maternal and neonatal outcomes.

**Study design:** Pregnant patients with ITP were enrolled in the study (platelet count <30×10^9^/L) between January 2015 and 2019. A total of 43 patients received LD oral prednisone (0.25–0.5 mg/kg) as the initial treatment and were compared retrospectively with the 31 patients in the HD (1 mg/kg) study. The primary clinical endpoint was the response rate, and the secondary endpoint was maternal hemorrhagic events, complications, and neonatal outcomes.

**Results:** In total, 35% of patients responded (15/43) to the LD cortico-therapy, including four patients with a complete response which was no less than HD therapy (35.5%). The bleeding symptoms of 10 (30%) patients were ameliorated after 14 days of LD prednisone treatment. Preeclampsia occurred in three cases (7% of total) of which the incidence was obviously lower than that of the previous study at HD (18%). No stillbirth or miscarriage occurred in the LD group, and neonatal outcomes had no significant differences between the two studies.

**Conclusion:** LD prednisone therapy for severe ITP patients during pregnancy had equal efficacy to HD treatment. In addition, the decrease in dosage significantly reduced the incidence of hypertension.

## Introduction

Immune thrombocytopenia (ITP) is an acquired autoimmune disease characterized by a transient or persistent decrease in platelet count, affecting approximately 1 in 1,000–10,000 pregnancies (Author Anonymous, 1999). Most women with ITP have mild to moderate thrombocytopenia, and 30%–35% require intervention during pregnancy. Similar to the treatment of ITP in a nonpregnant individual, both corticosteroids and intravenous immunoglobulin (IVIg) are the first-line treatments for ITP during pregnancy, and the *American Society of Hematology (ASH) 2011 evidence-based practice guideline for immune thrombocytopenia* recommends a starting dose of prednisone of 1 mg/kg daily (Neunert et al., 2011). However, our recent prospective clinical study (Xu et al., 2018) found that the response rate to prednisone at 1 mg/kg was not only lower than that reported in nonpregnant ITP patients (35.5% vs. 85%) but also increased the incidence of pregnancy-induced hypertension (22.2%). Therefore, in 2013, the ASH recommended a lower starting prednisone dose of 0.25 to 0.5 mg/kg daily (Anita et al., 2013). In 2019, the updated international report proposed that low-dose prednisone should be used as the initial treatment during pregnancy and then adjusted to the minimum dose (Provan et al., 2019). But evidence of whether the efficacy of a lower starting dose is nothing less than a higher dose (1 mg/kg) remains sparse. The aim of this study was to determine the safety and efficacy of low-dose prednisone therapy (defined as 0.25–0.5 mg/day) for the management of severe immune thrombocytopenia during pregnancy.

## Methods

### Study design

This was a prospective, observational study. The study design was approved by the Research Ethical Committee of Peking University People’s Hospital and supported by the Natural Science Foundation of Beijing.

### Patients

Pregnant women with ITP in Peking University People’s Hospital were invited to participate in this study. The confirmation and reconfirmation of the diagnosis of ITP were based on the international consensus report on the investigation and management of primary immune thrombocytopenia (Provan et al., 2010), including patients diagnosed before pregnancy and those newly diagnosed during pregnancy. Patients with a platelet count below 30×10^9^/L were enrolled in the study. Patients whose thrombocytopenia was secondary to infections (HIV, HCV, and *H pylori*), preeclampsia, and autoimmune disorders and those who developed other hematology or rheumatism diseases during pregnancy or postpartum follow-up were excluded. Patients whose pregnancies ended before 12 weeks were also excluded because of the difficulty of identifying and qualifying patients so shortly after the diagnosis of pregnancy and the high frequency of early miscarriage due to chromosome errors or unidentifiable causes. Informed consent was obtained from each patient in accordance with the Declaration of Helsinki.

Assessment of bleeding was performed at the time of enrollment, according to the reported scoring system by the ITP Working Party. Bleeding severity was graded as follows: grade 0, absence of bleeding; grade 1, petechiae; grade 2, ecchymoses and/or dripping with moderate loss of blood; grade 3, major mucous hemorrhage with copious loss of blood without sequelae; and grade 4, major mucous and/or parenchymal hemorrhage with copious loss of blood with sequelae and/or life-threatening or deadly.

### Methods

According to the consensus report on the management of immune thrombocytopenia during pregnancy from the Japanese Society of Hematology in 2014 (Miyakawa et al., 2014), we developed the following treatment plans:

• For patients enrolled in this study, the initial therapy is oral prednisone.
• The recommended starting dose of prednisone is 0.25 to 0.5 mg/kg daily.
• For patients not responding to prednisone, the alternative therapy is intravenous immunoglobulin (IVIg). The recommended starting dose of IVIg is 400 mg/kg.
• For patients refractory to initial single therapy, the combination of prednisolone and IVIg should be considered.

The primary endpoint of this study was the response rate on the 14th day after oral prednisone at a dose of 0.25–0.5 mg/kg. Complete response (CR) was defined as a maternal platelet count of at least 100×10^9^/L and the absence of bleeding. Partial response (PR) was defined as a platelet count of more than 30×10^9^/L and an increase of at least twice the baseline. Nonresponse was defined as a platelet count below 30×10^9^/L. Loss of response was defined as a platelet count fall below 30×10^9^/L or less than a two-fold increase from baseline after being responded.

We considered the following secondary endpoints: maternal hemorrhagic events include assessment of bleeding, prenatal hemorrhage, postpartum hemorrhage (>500 ml blood loss), and peripartum platelet transfusion; a maternal composite outcome of pregnancy-induced hypertensive (PIH) disorder, gestational diabetes (GDM), and infection and premature rupture of membranes (PROM); a fetal/neonatal composite outcome of the following adverse events: stillbirth, preterm birth before 37 weeks of gestation, small for gestational age size (birth weight, 10th percentile for gestational age), NICU admission, neonatal immune thrombocytopenia, or neonatal intracranial hemorrhage.

Considering both our previous data and other studies showed that the response rate of prednisone dose at 1 mg/kg was less effective in pregnancy and its potential adverse effect on blood pressure, ethically it is not suggested to carry out the case–control experiment at the same period. Therefore, this study will take previously published results by our center as a historical comparison (Xu et al., 2018).

The historical control group comprised 40 patients who received corticosteroid therapy at 1 mg/kg during pregnancy from January 2012 to October 2015, as per the standard protocol at that time. The primary outcome parameters were the response rate to prednisone and the incidence of maternal complications.

### Statistical analysis

Data were summarized using mean and standard deviation for quantitative variables and percentage for qualitative variables. In all cases, *p*-value < 0.05 was considered to be statistically significant. Fisher’s exact test and chi-squared test were used to evaluate the impact of clinical characteristics on the maternal and fetal outcome. Analysis was performed using SPSS 19.0.

## Results

### Patients

In total, 56 participants were enrolled in this study between January 2015 and January 2019. Among them, 43 patients (76%, 43/56) received oral prednisone as the initial treatment, while the remaining 13 patients were excluded because of refusing to take glucocorticoids after providing fully informed consent and chose intravenous infusion of gamma globulin instead. The median age of the 43 pregnant ITP patients was 30 years (interquartile range [IQR], 23–39 years), and 81.4% (35/43) were primigravidae. A total of 28 patients (65%) were diagnosed with ITP before pregnancy; 98% (42/43) of patients began low-dose cortico-therapy in the second and third trimesters of pregnancy, and only 1 case occurred at 7 weeks of gestation. The median gestational age at the time of enrollment was 28 weeks (IQR, 7–36 weeks), and the median baseline platelet count was 15×10^9^/L (IQR, 3–30×10^9^/L). Compared with the previous high-dose cortico-therapy group, there was no significant difference in the baseline characteristics of patients enrolled (Table 1).

**TABLE 1:** Baseline characteristics of patients taking prednisone enrolled in the study.

Characteristic	LD group (*n* = 43)	HD group (*n* = 40)*
Age, median (IQR), y	30 (23–39)	29 (24–38)
Gestational weeks, wk		
≤12^+6^, n (%)	1 (2.3)	0 (0)
13–19^+6^, n (%)	9 (20.9)	14 (35)
≥20, n (%)	33 (76.8)	26 (65)
Duration of corticosteroids, mean (SD), d	43.5 (23.2)	49 (28.9)
Platelet count on enrollment, median (IQR), ×10^9^/L	15 (3–30)	17 (6–29)
Primigravida, n (%)	35 (81.4)	24 (77.4)
Diagnosis of ITP, n (%)		
Before pregnancy	28 (65.1)	25 (62.5)
In pregnancy	15 (34.9)	15 (37.5)
BMI, mean (SD) kg/m^2^	22.03 (2.77)	22.24 (3.4)

* Data in the HD (high-dose) group were obtained from our previous study (Xu et al., 2018); LD: low-dose.

### General characteristics and response of low-dose cortico-therapy

**FIGURE 1:**
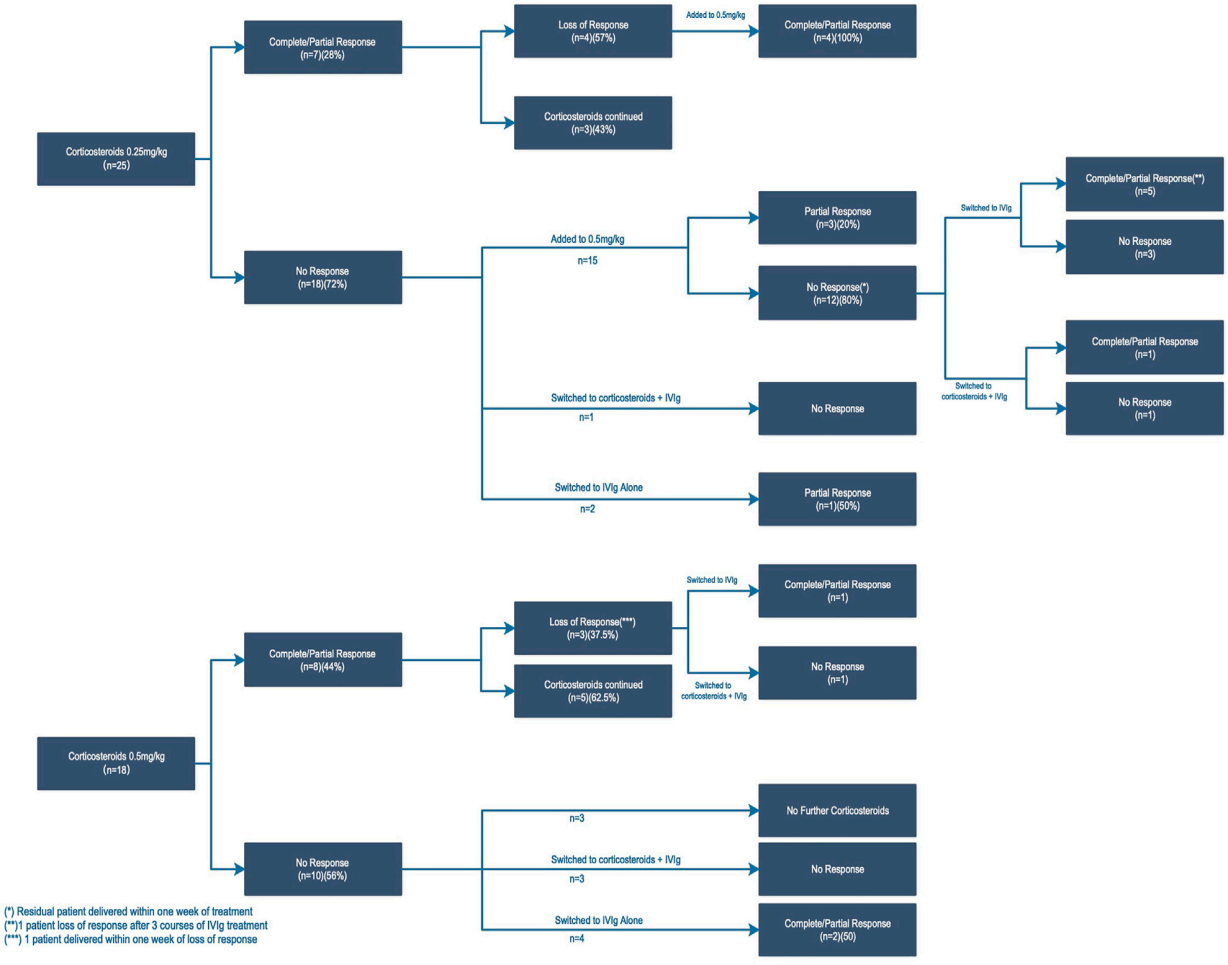
Response rate according to treatment strategies for individuals.

The treatment procedures and response to platelet count of 43 patients who received oral prednisone at an initial dose of 0.25–0.5 mg/kg are presented in Figure 1. Here, 35% of patients responded (15/43) to low-dose cortico-therapy, including 4 patients with complete response and 11 patients with partial response. The increase in the platelet count was measured at a median (IQR) of 7 (7–28) days following initiation of prednisone. The time of prednisone initiation during pregnancy has no impact on the response rate (33.3% in second trimesters vs. 36.4% in third trimesters). Similarly, there was no significant difference in the response rate between patients with ITP diagnosed before and after pregnancy.

Response occurred in 7/25 (28%) pregnancies treated initially with 0.25 mg/kg of prednisone and 8/18 (44%) pregnancies with 0.5 mg/kg (*p* = 0.26). Four responders (57.1%) with an initial dose of 0.25 mg/kg showed a loss of response after 4–8 weeks, but they all responded again when the prednisone dose was increased to 0.5 mg/kg. In the majority of non-response patients to 0.25 mg/kg, a lack of response in the platelet count persisted despite increasing to 0.5 mg/kg (12/15, 80%) (Figure Figure1). However, there were 15 non-response patients to prednisone who switched to IVIg treatment, of which 46.7% (7/15) responded. Six refractory ITP patients (defined as non-response to prednisone and/or IVIg) underwent combination therapy (methylprednisolone plus IVIg), and the response rate was 33.0% (2/6). As a summary of the aforementioned results, the comprehensive efficiency of our low-dose initial glucocorticoid therapy strategy is 55.8% (24/43).

### Maternal and neonatal outcomes

It is noticed that the majority of the patients enrolled in this study had no or minor bleeding tendency before treatment despite a severe reduction in platelet counts, which may be associated with the hypercoagulable state of pregnancy. The bleeding tendency of most people is mainly easy bruising or purpura and ecchymosis. Only a few patients exhibited epistaxis, and one patient had hematuria. It appears that the bleeding symptoms of 10 (30%) patients were ameliorated after 14 days of prednisone treatment (Table 2).

**TABLE 2:** Bleeding score before and after prednisone treatment.

Bleeding score	Enrollment, n (%)		Day 14, n (%)	
	LD group	HD group	LD group	HD group
0	30 (69.8)	27 (67.5)	37 (86.0)	35 (87.5)
1	7 (16.2)	9 (22.5)	5 (11.6)	4 (10)
2	3 (7.0)	2 (5)	1 (2.3)	1 (2.5)
3	2 (4.6)	1 (2.5)	0 (0)	0 (0)
4	1 (2.3)	2 (5)	0 (0)	0 (0)

LD: low-dose; HD: high-dose.

**FIGURE 2:**
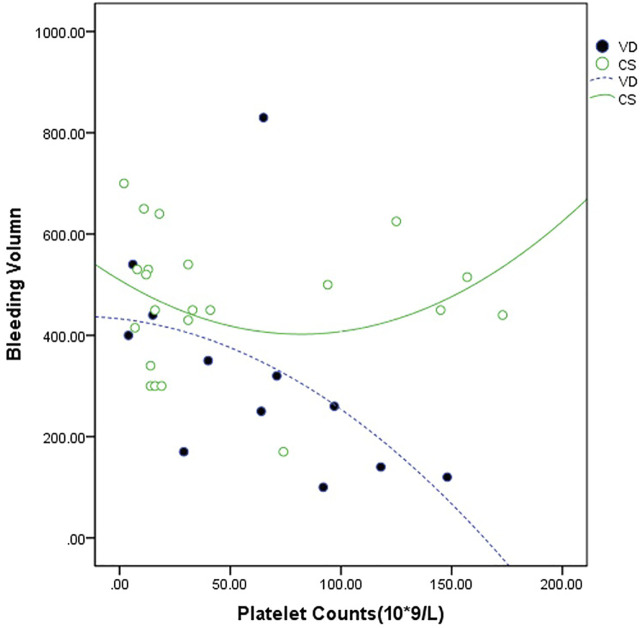
Relationship between the maternal platelet count at delivery and the bleeding volume. The bleeding volume was measured in 14 cases of vaginal delivery (VD --•--) and 29 cases of the Cesarean section (CS **—**○**—**).

Infants were born by vaginal delivery in 14 cases (32.6%) and by cesarean delivery in 29 cases (67.4%). After eliminating 7 cases with Cesarean section indications, including funnel pelvis, scarred uterus, breech presentation, and twins, the proportion of cesarean delivery could still reach 51%. For patients of all platelet counts, the bleeding volume was greater in Cesarean deliveries than in vaginal deliveries. In Cesarean delivery patients, the bleeding volume did not diminish in the proportion to the increased platelet count. On the other hand, in vaginal delivery patients, even patients with a platelet count less than 50×10^9^/L, no bleeding volume over 1,000 ml occurred (Figure 2).

Complications occurred in 19 cases (44%) during pregnancy (Table3). GDM occurred in 9 cases (21% of total), and this incidence was higher than that among total pregnancies in China (10%). However, there were three patients who were diagnosed before cortico-therapy through an OGTT test. Preeclampsia occurred in 3 cases (7% of the total), of which the incidence was obviously lower than that in our previous study at a prednisone dose of 1 mg/kg (18%).

**TABLE 3:** Maternal outcome of 43 women with low-dose prednisone.

	Total
PIH, n (%)	3 (7%)^a^
GDM, n (%)	9 (21%)
PROM, n (%)	6 (14%)
Infection, n (%)	1 (2%)
Prenatal hemorrhage, n (%)	3 (7%)
Preterm birth, n (%)	8 (19%)
Mode of delivery, n (%)	
Vaginal delivery	14 (33%)
Cesarean section	29 (67%)
Onset of labor, n (%)	
Spontaneous	5 (29%)
Induced	12 (71%)
Platelets at delivery, median (IQR),×10^9^/L	31 (2–173)
Gestational week at delivery	37.6 ± 1.4
Postpartum hemorrhage	23 (53%)
Blood loss, median (IQR),ml	500 (100–1,500)
≥1,000 ml,n	3
Platelet transfusion (U)	1.4 ± 0.2

^a^ The incidence was 18% in the previous study at a prednisone dose of 1 mg/kg.

For these 43 pregnancies (44 fetuses, 1 set of twins), no stillbirth or miscarriage occurred. The median birth platelet count was 256×10^9^/L (range 8–440). Nine babies (21%) had platelet counts <150 × 10^9^/L at birth (Table 4). Four babies received treatment for thrombocytopenia, and there were no cases of intracranial hemorrhage or neonatal deaths. The remaining five infants were reported to have spontaneous recovery of their platelets. There were eight premature infants whose gestational weeks were less than 37 weeks but more than 34 weeks. All the preterm births occurred in refractory ITP patients, and only 1 case benefited from combination therapy. Four premature labors were due to premature rupture of membranes, and the remaining 4 cases were all iatrogenic preterm labors due to the ineffective treatment of severe thrombocytopenia. The incidence rates of these complications were significantly higher in the poor clinical state group (platelet count <30×10^9^/L) than in the good control group (platelet count>30×10^9^/L) (*p* = 0.036).

**TABLE 4:** Neonatal outcomes for 44 infants of 43 women with low-dose.

	Total
Miscarriage	0
Stillbirth	0
Birthweight, g	3201 ± 402
LBW, n (%)	3 (7%)
FGR	0
Neonatal thrombocytopenia	9 (21%)
Platelet nadir	—
<20 × 10^9^/L	1
20–50 × 10^9^/L	3
51–100 × 10^9^/L	3
100–150 × 10^9^/L	2
Admissions to NICU	12 (27%)
Intracranial haemorrhage	0

## Discussion

It is now generally agreed that therapy for pregnant mothers with ITP is not similar to that for nonpregnant patients. Outside of pregnancy, corticosteroids have been shown to induce an initial response of up to 60%–80% in ITP patients (Nicolescu et al., 2013). However, despite using a higher dose of prednisone (1 mg/kg), both Sun et al. (2016) and our previous prospective study (Xu et al., 2018) confirm that the response rate of cortico-therapy in ITP patients during pregnancy is only 34%–38%. Although a starting prednisone dose of 0.25–0.5 mg/kg daily was recommended by some experts, such a low response rate raises concern that a lower corticosteroid dose is ineffective in this setting. Therefore, it is necessary to establish procedures based on evidence for low-dose prednisone therapy of ITP during pregnancy. Our study is the first to determine the efficiency of low-dose prednisone in the management of ITP during pregnancy, and it turns out that the response rate of lower-dose prednisone is not less than that of the higher dose. Although loss of response exists in the patients with an initial dose of 0.25 mg/kg, the same patients responded again after the dose was increased to 0.5 mg/kg. For patients with no response to low-dose cortico-therapy, it seems that other treatments are also ineffective. Eventually, the final response rate in this study is up to 55.8% (24/43), and this result is consistent with our previous study.

However, the use of low-dose prednisone therapy in patients with a bleeding tendency is still controversial. The bleeding tendency of all the patients in the study was not serious. Only one patient had a bleeding score of 4 points. Hypercoagulation in pregnant women may contribute to the prevention of massive bleeding with spontaneous mechanical trauma. Japanese researchers believe that the course of corticosteroid treatment should be determined based on the bleeding tendency, rather than platelet counts (Miyakawa et al., 2014). However, when the bleeding tendency is obvious, IVIg treatment is still recommended to achieve a safe platelet count quickly.

The benefits of reducing the dose of cortico-therapy are obvious. Although relatively safe in pregnancy, prednisone can increase weight gain, induce hyperglycemia, exacerbate hypertension, and contribute to adverse pregnancy outcomes. A case–control study by Martel et al. (2005) indicated that corticosteroids significantly increase the incidence of gestational hypertension and preeclampsia (OR 1.57 and 1.72, respectively), and a nationwide retrospective survey from a total of 284 pregnant women with ITP indicated that the occurrence of maternal complications such as preeclampsia differed significantly in the large-dose corticosteroid (≥15 mg/day) group compared with the untreated group (33.6% vs 21.6%, *p =* 0.049) (Fujimura et al., 2002). Our previous study (Xu et al., 2018) showed an 18% incidence of pregnancy-induced hypertensive disorders at a prednisone dose of 1 mg/kg, whereas the incidence was 7% at a prednisone dose of 0.25–0.5 mg/kg. In the previous study, more than half of the patients who were concerned about the long-term use of high-dose prednisone in pregnancy prefer IVIg. Although available evidence suggests there is no clear advantage of one agent over the other, cost is a problem that patients in developing countries have to consider. In this study, 76% of the severe thrombocytopenia patients with therapeutic indications during the same period preferred prednisone as their initial treatment after reducing the dosage, which significantly reduced the treatment courses and expensive cost of gamma globulin.

The rates of postpartum hemorrhage (7%) and preterm birth (19%) in this study were lower than those in the previous higher-dose cortico-therapy study (Xu et al., 2018) (51% and 45%, respectively). Improved healthcare standards in our center over these years can be a factor. In this study, the proportion of Cesarean sections persisted high. This is likely to be related to most of the patients’ platelet count still being difficult to reach 50×10^9^/L before delivery. Because of the suspicion of the safety of vaginal delivery, a Cesarean section is required. In fact, none of the patients who chose vaginal delivery despite their platelet count being less than 50×10^9^/L, or even less than 30×10^9^/L, experienced serious postpartum hemorrhage in this study. Moreover, the fitted curve shows when the platelet count was less than 50×10^9^/L, and the volume of blood loss at Cesarean delivery was greater than that at vaginal delivery. On the other hand, the amount of bleeding decreased gradually with the increase in platelet count, but the Cesarean section did not follow this rule. These findings suggest that we should consider patients whose platelet count is below 50×10^9^/L in the perinatal period in order to further improve the platelet count and reduce the amount of bleeding. At the same time, the choice of the delivery pattern in ITP patients should be based on obstetric factors and should not be used as a basis for vaginal delivery.

Thrombocytopenia was found in 14% of neonates, of which two newborns were delivered by vaginal and three were by Caesarean birth. However, there were no neonates with severe bleeding such as cerebral bleeding, and most infants recovered to a normal platelet count spontaneously within 1 month. This incidence of thrombocytopenia was similar to those described previously. Consistent with previous reports, there were no neonatal deaths in this study, and fetal/neonatal outcomes were overall favorable. The therapeutic effect is still the most critical factor determining the birth weight and gestational age of newborns.

The main limitation of our study is its sample size. Due to the low morbidity of ITP during pregnancy, despite being a referral center in East China, we still need to conduct a multi-center study to further expand the sample size. For ethical reasons, it is difficult to achieve a completely randomized controlled study. As in all non-randomized studies, our results may be impacted by the selection bias, and the historical control increases various biases compared to the parallel control. However, this study reflects the true situation of the patient population and external authenticity.

## Conclusion

This study suggests that lower-dose prednisone treatment in severe immune thrombocytopenia during pregnancy is not less effective than higher-dose prednisone treatment, and it reduces the incidence of maternal complications. Our work has provided evidence for the application of lower-dose glucocorticoid in ITP during pregnancy and paved the way for further multicenter, randomized controlled clinical studies.

## Data Availability

The original contributions presented in the study are included in the article/Supplementary Material; further inquiries can be directed to the corresponding author.
