# Supplementary material for: Lower-dose corticosteroid therapy in severe immune thrombocytopenia during pregnancy: The comparable efficacy and lower incidence of maternal complications
Source: Front Pharmacol. 2022 Oct 20;13:983734. doi: 10.3389/fphar.2022.983734 (PMC9633254; doi:10.3389/fphar.2022.983734)
Supplement: Supplementary file 1 [file Table1.DOCX]

Supplementary Material

# Supplementary Tables

Table S1 Maternal outcome according to diagnosis time of ITP

|  | Diagnosed before pregnancy  (n=28) | Diagnosed during pregnancy  (n=15) |
| --- | --- | --- |
| Response rate, (%) | 35.7 | 33.3 |
| PIH, n (%) | 3(10.7) | 0(0) |
| GDM, n (%) | 5(17.9) | 4(26.7) |
| PROM, n (%) | 4(14.3) | 2(13.3) |
| Infection, n (%) | 0(0) | 1(6.7) |
| Prenatal Hemorrhage, n (%) | 2(7.1) | 1(6.7) |
| Preterm Birth, n (%) | 5(17.9) | 3(20) |
| Mode of Delivery, n (%) |  |  |
| Vaginal Delivery | 8(28.6) | 6(40) |
| Cesarean Section | 20(71.4) | 9(60) |
| Platelets at Delivery, median (IQR),×10^9^/L | 26.5(2-125) | 41(6-157) |
| Gestational Week at Delivery | 37.5±1.2 | 37.7±1.7 |
| Postpartum Hemorrhage, n (%) | 16(57.1) | 7(46.7) |
